# Computational Analysis of Intravascular OCT Images for Future Clinical Support: A Comprehensive Review

**DOI:** 10.1109/RBME.2025.3530244

**Published:** 2026

**Authors:** Juhwan Lee, Yazan Gharaibeh, Pengfei Dong, Luis A. P. Dallan, Gabriel T. R. Pereira, Justin N. Kim, Ammar Hoori, Linxia Gu, Hiram G. Bezerra, Bernardo Cortese, David L. Wilson

**Affiliations:** Department of Biomedical Engineering, Case Western Reserve University, Cleveland, OH 44106 USA; Department of Biomedical Engineering, The Hashemite University, Zarqa 13133, Jordan; Department of Biomedical Engineering and Sciences, Florida Institute of Technology, Melbourne, FL 32901 USA; Harrington Heart and Vascular Institute, University Hospitals Cleveland Medical Center, Cleveland, OH 44106 USA; Harrington Heart and Vascular Institute, University Hospitals Cleveland Medical Center, Cleveland, OH 44106 USA; Department of Biomedical Engineering, Case Western Reserve University, Cleveland, OH 44106 USA; Department of Biomedical Engineering, Case Western Reserve University, Cleveland, OH 44106 USA; Department of Biomedical Engineering and Sciences, Florida Institute of Technology, Melbourne, FL 32901 USA; Heart and Vascular Institute, University of South Florida, Tampa, FL 33606 USA; Harrington Heart and Vascular Institute, University Hospitals Cleveland Medical Center, Cleveland, OH 44106 USA; Department of Biomedical Engineering and Radiology, Case Western Reserve University, Cleveland, OH 44106 USA

**Keywords:** Artificial intelligence, deep learning, finite element modeling, intravascular optical coherence tomography, machine learning

## Abstract

Over the past two decades, intravascular optical coherence tomography (IVOCT) has emerged as a promising tool for planning percutaneous coronary interventions (PCI), studying coronary artery disease, and assessing treatments. With its near-histological resolution and optical contrast, IVOCT uniquely evaluates coronary plaque characteristics, enhancing the guidance of interventional procedures. Artificial intelligence (AI) techniques have been widely applied to IVOCT imaging, providing fast and accurate automated interpretation. These techniques hold significant potential for both clinical and research purposes. Clinically, automated analysis offers comprehensive assessments of coronary plaques, leading to better treatment decisions during PCI. For research, automated interpretation of IVOCT opens new avenues to understand the pathophysiology of coronary atherosclerosis. However, these techniques face several limitations, including issues related to spatial resolution, challenges in manual assessments, and the additional time required for these analyses. This review covers recent advancements and applications of AI techniques and computational simulation methods in IVOCT image analysis, including vessel wall segmentation, plaque characterization, stent analysis, and their clinical applications. Furthermore, we discuss the potential of AI-enhanced IVOCT analysis to facilitate personalized decision-making, potentially improving short- and long-term patient outcomes.

## I. Introduction

CARDIOVASCULAR disease is the leading cause of death in the United States, with coronary artery disease being the most prevalent. Percutaneous coronary intervention (PCI) is the most common coronary revascularization procedure, accounting for over 600,000 cases per year in the US [1]. This number increases further when considering peripheral artery, carotid artery, and renal stents. Non-invasive imaging techniques like X-ray angiography and computed tomography (CT) angiography primarily reveal the coronary arteries’ lumen, providing limited insight into arterial wall tissues. The principal methods for intracoronary imaging are intravascular optical coherence tomography (IVOCT) and intravascular ultrasound (IVUS). These modalities are widely used to assess plaque characteristics, guide PCI, and evaluate stent deployment and apposition. Additionally, IVUS-combined with near-infrared spectroscopy has been clinically utilized to aid interventional procedures. One of IVOCT’s primary advantages over IVUS is its tenfold greater spatial resolution, enabling the visualization of intimal thickening below the detection threshold of IVUS [2]. Numerous studies have demonstrated IVOCT’s superior accuracy in measuring plaque distribution compared to IVUS [3], [4], [5]. Notably, our institution, University Hospitals of Cleveland in Cleveland, OH, was one of the first hospitals in the US to use IVOCT for clinical purposes in 2010.

Over the past decade, IVOCT has become a promising tool for planning PCI. IVOCT-guided PCI offers significant benefits over X-ray angiography-guided PCI alone [6], [7]. With its near-histological resolution and optical contrast, IVOCT uniquely assesses plaque characteristics, enhancing the ability to evaluate plaque vulnerability. IVOCT is particularly recognized as one of the best methods for identifying high-risk local lesions, such as thin cap fibroatheroma (TCFA) and microchannel [8]. More than 20 histological and clinical studies have conclusively shown that IVOCT can assess in vivo histological plaque characteristics, including lipid/calcium [9], [10], [11], fibrous cap [12], [13], [14], [15], [16], macrophage presence [14], [17], cholesterol crystals [18], [19], microchannels [20], [21], thrombus [22], [23], [24], and intraplaque hemorrhage [25], [26], [27]. These histological findings, observable via IVOCT, have been linked to risk in various pathology studies [9], [10], [11], [12], [13], [14], [15], [16], [17], [18], [19], [20], [21], [22], [23], [24], [25], [26], [27]. Consequently, the enhanced clinical capabilities of IVOCT have led to its increased application in PCI, the most widely performed revascularization treatment for coronary heart diseases.

Automated interpretation of IVOCT using advanced image analysis techniques, particularly those involving machine and deep learning, holds significant potential for both clinical and research purposes. For clinical purposes, fast and accurate automated analysis can provide comprehensive assessments of coronary plaques, aiding in the selection of the best treatment plan during PCI. Moreover, it can predict stenting outcomes and OCT-derived fractional flow reserve (FFR), helping to avoid adverse outcomes [28], [29]. For research purposes, automated interpretation of IVOCT opens new opportunities by enabling detailed examination of plaque structures at the microscopic level. This can deepen our understanding of the pathophysiology of coronary atherosclerosis, linking numerous high-risk plaque features to short- and long-term clinical outcomes. Additionally, the integration of artificial intelligence (AI)-driven analysis with large datasets can enhance the precision of these predictions, ultimately contributing to more personalized and effective treatments.

This report provides an up-to-date review of the clinical applications of IVOCT in the quantitative assessment of coronary plaques, focusing on newer technologies and their role in future clinical support. Our review primarily addresses issues related to automated image analysis, such as plaque characterization and stent analysis, and their clinical applications for predicting short- and long-term adverse outcomes.

## II. Search Criterion

**Fig. 1.**
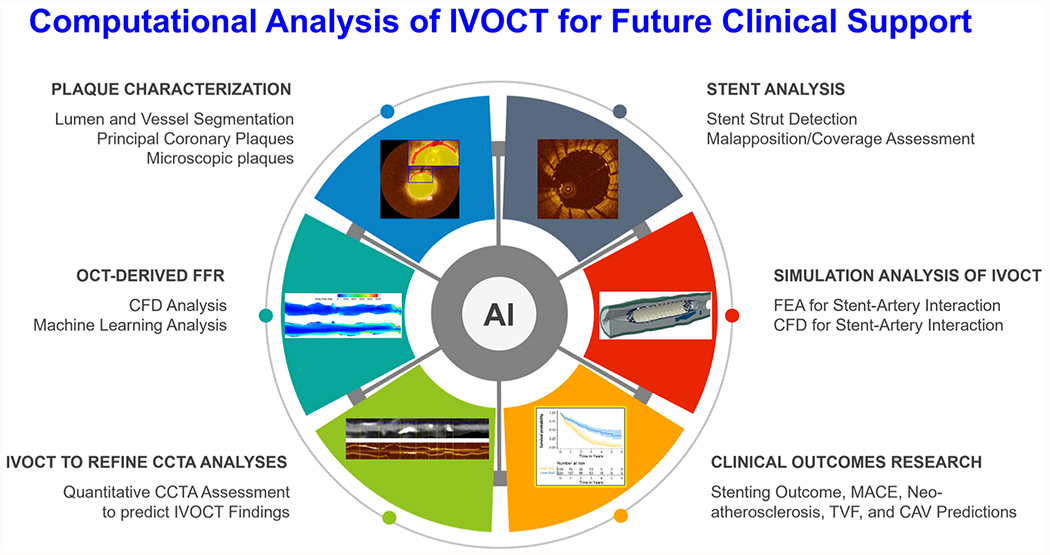
Diagram illustrating the computational analysis of IVOCT for future clinical support.

To identify related contributions, we conducted search in the electronic databases such as PubMed, EMBASE, and Google Scholar for all published studies that applied advanced image analysis techniques in IVOCT images. The following keywords in different combinations were used: intravascular optical coherence tomography, quantitative, image processing, AI, machine learning, deep learning, finite element modeling, computational fluid dynamics, simulation, coronary artery wall, plaque, atherosclerosis, FFR, registration, stenting outcome, and clinical outcome. The literature search mainly focused on studies published after 2018 and up to April 2024. Although there were some image analysis papers prior to 2018, most were limited with regards to automation. Only the peer reviewed papers were included, while no conference proceedings, abstracts, preprint (e.g., arXiv and medRxiv), and case reports were included. Fig. 1 provides a summary of the computational analysis of IVOCT for future clinical support.

## III. Plaque Characterization

### A. Lumen and Vessel Segmentation

Segmentation of the IVOCT lumen and vessel (i.e., lumen, intima, media, and adventitia) is a crucial step in the quantitative assessment of arterial morphology. For example, lumen segmentation can be utilized to calculate the severity of stenosis and lipid/calcification angle. However, manual tracing of the vessel in IVOCT images is labor-intensive and time-consuming due to the large number of images (up to 540) acquired during a typical IVOCT examination. Consequently, automated methods based on recent machine/deep learning techniques could significantly enhance the efficient analysis of coronary artery disease. This is clinically relevant as a robust indicator for selecting the optimal stent landing zone. In addition, this can be used to numerous research purposes associated with the progression of pathological formations and future risk. Automated vessel segmentations can be divided into two main approaches: image processing-based and learning-based. Supplementary Table S1 summarizes these studies on automated vessel segmentation, as well as downstream analyses.

#### 1) Image Processing-Based Lumen/Vessel Wall Segmentation:

**Fig. 2.**
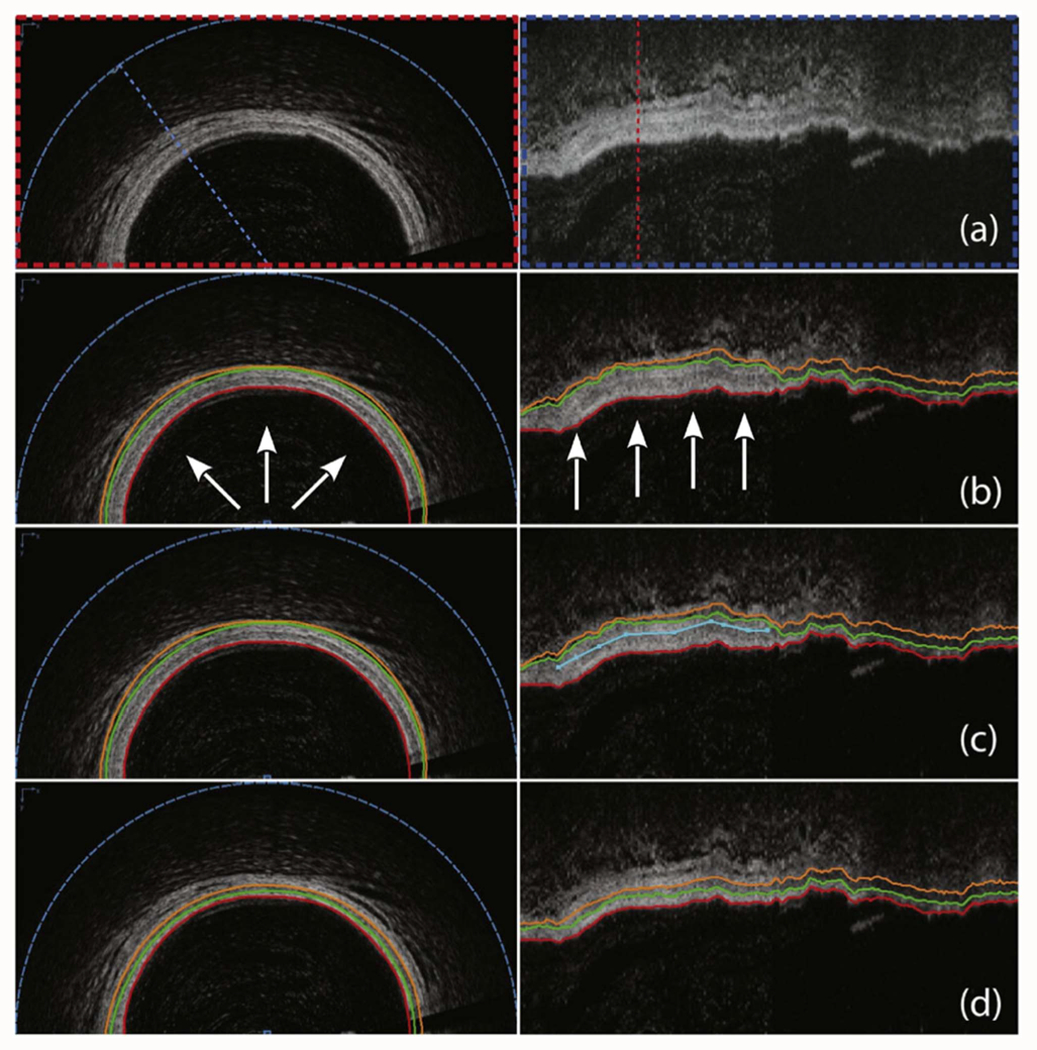
Automated OCT segmentation followed by JEI yielding clinically acceptable 3D segmentation of coronary wall layers. (a) Original cross-sectional and axial views of a 3D OCT dataset. (b) Automated 3-surface 3D LOGISMOS approach shows a regional segmentation inaccuracy (arrows) with lumen in red, outer intima in green and outer media in orange. (c) JEI interactions shown in turquoise color provide a suggested position for the outer media (orange) surface in the axial view. (d) Multi-surface 3D segmentation is re-optimized every time a set of correction points is provided –thefew identified points shown completely corrected the inaccuracy in 3D. Note that all JEI modifications are optional such that the full segmentation workflow can be completed either without any interaction (fully automated) or using human expertise to guide the segmentation via JEI when needed. Important to realize is that the experts interact with the algorithm, they never directly retrace the borders in the image.

There have been multiple publications using image processing approaches without the benefit of a learning system. For example, one study [30] employed the Layered Optimal Graph-based Image Segmentation for Multiple Objects and Surfaces (LOGISMOS) approach for segmenting wall-layers to assess the progression of cardiac allograft vasculopathy (CAV) (Fig. 2). This method demonstrated a high correlation (*R^2^* = 0.93) between automated and manual segmentations in measuring intimal + medial thickness, with an average error of 4.98 *±* 31.24 *μm*, aligning closely with inter-observer variability. Another research effort [31] introduced automatic lumen segmentation by detecting a flat structuring element and applying linear interpolation. When tested on 667 image frames, the algorithm achieved an interclass correlation of 0.97 in lumen area assessments, indicating a high level of agreement with manual methods. Olender et al. [32] proposed an anisotropic linear-elastic mesh method to delineate vessel walls in OCT images. Validated on 724 frames from seven patients, the method achieved an R^2^ of 0.89 for cross-sectional area comparisons. It outperformed conventional interpolation techniques, providing smoother and more accurate outer vessel border reconstructions for diseased vessels. Furthermore, an automated process for extracting lumen contours, incorporating pre-processing, interpolation, and morphological dilation-erosion smoothing, was developed [33]. This method, evaluated on 5,931 pre-stent IVOCT images, demonstrated a strong correlation (*R* = 0.988) with manual labeling. Additionally, it showed a strong correlation (*R* = 0.978) in OCT-derived FFR comparisons. Another innovative approach [34] for segmenting lumen borders utilized a unique feature calculation approach consisting of a gradient convolution field and a Laplacian diffusion. This method achieved a sensitivity of 96.4% and an F1 score of 97.6% compared to manual assessments, highlighting its accuracy and reliability. Additionally, leveraging the uniqueness of vascular wall connected region on A-lines, another study [35] effectively segmented the vessel lumen in IVOCT images. The dataset included 1,436 image frames from six patients with intermediate stenosis and various intensity artifacts. They implemented a series of preprocessing steps (e.g., Gaussian filtering, binarization, morphological operation, catheter removal) and a double lumen contour restoration scheme, which showed superior performance compared to traditional approaches like dynamic programming. Most recently, an automatic method for multilayer segmentation in constructing biomechanical vessel models was developed [36]. Utilizing IVOCT data from six patients, they performed preprocessing, lumen detection using Otsu’s thresholding [37], and layer edge detection using the Canny edge and cubic spline surface fitting. The average errors of the automatic contours compared to manually segmented contours were 1.40% (internal elastic membrane), 4.34% (external elastic membrane), and 6.97% (adventitia-periadventitia interface).

#### 2) Learning-Based Lumen/Vessel Wall Segmentation:

Learning-based algorithms, categorized into traditional machine learning [38] and supervised deep learning [39], [40], have greatly improved the segmentation accuracy of the lumen and vessel wall in IVOCT images. Building on their previous study [41], one research group [38] utilized a traditional machine learning model to diagnose Kawasaki disease using IVOCT imaging. Their study included 5,040 image frames, consisting of 2,900 normal frames and 2,140 pathological frames. They trained a random forest model with pre-trained VGG-19 features to differentiate between normal and diseased arterial walls. Additionally, a VGG-19-based fully convolutional network (FCN) was employed to detect tissue layers in normal cases. This method demonstrated high effectiveness with a sensitivity of 0.93 for intima and 0.91 for media, and an accuracy of 0.90 for intima and 0.87 for media.

For supervised deep learning, Gharaibeh from our group [39] segmented the vascular lumen using a refined pre-trained SegNet, followed by cleaning the segmentation results with conditional random field (CRF) technique. Their approach enhanced data by concatenating polar IVOCT images and shifting the start point, analyzing 48 volumes from 34 clinical pullbacks. After CRF processing, the method achieved a segmentation sensitivity of 0.99 and a Dice coefficient of 0.98. They also argued that using the raw polar (*r,θ*) data shows better segmentation performance than using the Cartesian (*x,y*) data. Additionally, a new tool called DeepCap [40], based on a U-Net architecture, was introduced for automated vessel lumen segmentation, optimized for memory efficiency. This study included 12011 expert-labeled images from 22 patients, encompassing diverse image conditions such as blood and light artifacts, as well as metallic and bioresorbable stents. DeepCap demonstrated notable efficiency, utilizing only 12% of the parameters of other models and chieving up to 70% faster per-image inference times on GPUs.

### B. Identification of Principal Coronary Plaques

Coronary plaque identification is important both for research and for planning PCI procedures. When treating highly calcified coronary artery lesions with stents, interventional cardiologists make stressful treatment decisions that can lead to inadequate stent deployment. Additionally, the severity of calcification is strongly associated with the degree of atherosclerosis, and the extent of calcification is linked to higher complication rates during or after PCI [42]. Moreover, the extent of lipidic plaque is critical to consider, particularly for research studies such as drug development, and to avoid placing the stent edge over a lipidic lesion, especially in cases of TCFA, due to its instability and potential to promote adverse vascular responses. Therefore, accurate identification of coronary plaques is essential for both plaque-specific lesion preparation and the appropriate selection of therapeutic strategies during PCI [43], [44]. Recent image processing methods for coronary plaque characterization can be divided into frame-level plaque classification, A-line classification, and pixel-wise classification. Supplementary Table S1 summarizes the automated principal plaque segmentation studies.

#### 1) Frame-Level Plaque Classification:

Frame-based methods typically classify each IVOCT frame as either normal or diseased (e.g., calcified). Although these methods do not provide quantitative plaque assessments, they still offer clinically meaningful information. All existing plaque classification studies have been based on supervised deep learning approaches. For instance, one study [45] employed a two-path deep learning architecture to classify IVOCT images as plaque or non-plaque. They used data augmentation techniques, training two separate ResNet50-V2 models on Cartesian and polar images. Their combined model achieved an accuracy of 91.7%, sensitivity of 90.9%, and specificity of 92.4%, demonstrating strong plaque classification capabilities. Another study [46] developed a ResNet-based 3D deep learning network to classify IVOCT frames as either calcification positive or negative. They initialized the model with a pretrained ResNet-50 network and retrained it on approximately 4860 images. To address data imbalance, they employed a two-phase training approach, achieving a precision of 96.9%, sensitivity of 97.7%, and an F1 score of 96.1%, showcasing the efficacy of CNN models in plaque classification. Additionally, an automated diagnostic algorithm for assessing plaque vulnerability in IVOCT images was introduced [47]. Using a DenseNet-121 model trained on 44947 pre-labeled images, their algorithm achieved a diagnostic accuracy of 94.0%, outperforming the average accuracy of 83.8% among four general cardiologists. The algorithm’s AUCs for distinguishing normal vessel, stable plaques, and vulnerable plaques were 0.992, 0.952, and 0.998, respectively. Moreover, for the first time, a Vision Transformer (ViT)-based deep learning model [48] was utilized to detect layered plaque in IVOCT images [49]. This study employed the largest dataset to date with over 300,000 images (237021 for training/validation and 65394 for external validation). The ViT-based model outperformed a standard CNN-based model, achieving an AUC of 0.860 compared to 0.799, indicating the potential of ViT models in medical imaging analysis.

##### 2) A-Line Classification:

A-line classification methods focus on each A-line along with their adjacent A-lines in the raw polar (*r,θ*) IVOCT images. Unlike pixel-wise classification, A-line classification considers each A-line as containing mixed plaques, such as fibrous and lipidic or fibrous and calcified, and typically classifies the plaque as fibrolipidic and fibrocalcific. Previous studies can be categorized into three groups, consisting of traditional machine learning-based, CNN-based, and hybrid approaches. For machine learning, Prabhu from our group [50] utilized a comprehensive set of handcrafted features, including lumen morphology and optical attenuation, to identify fibrolipidic and fibrocalcific A-lines. They developed a machine learning model using random forest and support vector machine (SVM), trained on an extensive dataset of approximately 7000 images, including clinical and ex-vivo images, and validated against 3D cryo-imaging/histology. CRF was used for 3D classification noise cleaning. On a test set of over 1700 images, the model achieved sensitivities/specificities of 94.5%/87.3% for fibrolipidic and 74.8%/95.3% for fibrocalcific plaques.

For CNN-based approaches, another research from our group [51] developed an automated method for plaque classification. Their preprocessing steps included lumen segmentation, guidewire shadow removal, and noise reduction. The CNN architecture featured two convolutional, two max-pooling, and two fully connected layers. After classification, they applied a CRF and morphological processing to clean noisy A-line classifications. This CNN approach achieved accuracies of 77.7% for fibrocalcific, 86.5% for fibrolipidic, and 85.3% for other plaque types. Notably, CRF cleaning improved classification results by 10–15%. Additionally, one study [52] classified A-lines in IVOCT as fibrolipidic or fibrocalcific plaques using a shallow CNN architecture. They first identified the lumen boundary using the ARC-OCT algorithm [53] and then classified each A-line with a CNN model comprising three convolutional and two fully connected layers. Classification accuracy was further enhanced by incorporating OCT-specific transformed images based on attenuation coefficient estimation, improving the over-all accuracy from 74.9% to 83.5%.

For a hybrid approach, another innovative method from our group [54] combined deep learning features with handcrafted lumen morphology features for plaque characterization in IVOCT images. They extracted 100 deep learning convolutional features and 371 lumen morphological (e.g., eccentricity, best-fit line features, and superficial attenuation) features, training a random forest classifier on these 471 features. Their approach achieved sensitivities/specificities of 84.8%/97.8% and 91.2%/96.2% for fibrolipidic and fibrocalcific plaques, respectively, indicating a promising result in plaque characterization.

###### 3) Pixel-Wise Classification:

Pixel-wise classification provides the most comprehensive assessment of plaque morphology by analyzing each pixel in IVOCT images. All existing pixe-wise classification studies have been conducted using supervised deep learning approaches. For instance, one method [55] combined CNN and the random walk algorithm for plaque segmentation. The CNN pre-segmented the plaque area, providing seed points for the random walk refinement. The Jaccard similarity coefficients for lipid, calcified, and fibrotic plaques were 0.864, 0.864, and 0.876, respectively, showing high similarity to manual assessments. Another approach [39] employed a pretrained SegNet model for vascular lumen and calcification segmentation. This method involved preprocessing steps (e.g., log transformation and noise reduction) and data augmentation by concatenating raw polar IVOCT images with offset angular shift. It achieved sensitivities of 0.85, 0.99, and 0.97 for calcified, lumen, and other tissues, respectively. Agreement between manually and automatically obtained stent-deployment calcification scores [28] was acceptable. Additionally, a fully automated semantic segmentation method using SegNet and CRF for noise reduction was developed by our group [56]. After preprocessing steps, including lumen segmentation, guidewire shadow removal, pixel shifting, and noise filtering, the SegNet model was trained on 4,892 image frames across 57 pullbacks. Their method achieved sensitivities, specificities, and Dice coefficients of 87.4%/89.5%/0.801 for lipidous plaques and 85.1%/94.2%/0.734 for calcified plaques. The differences in mean clinical plaque attributes (e.g., arc angle and depth) between manual and automated methods were minimal (<4%). A novel approach [57] introduced a deep residual U-Net for vulnerable plaque segmentation, employing a loss function that combined weighted cross-entropy and the Dice coefficient. To address class imbalance, the original IVOCT data was augmented by rotating the image foreground clockwise by 30–300 degrees. This method was evaluated using pixel accuracy, mean pixel accuracy, mean intersection over union, frequency weight intersection over union, precision, and sensitivity, outperforming conventional models with a sensitivity of 91.4%.

**Fig. 3.**
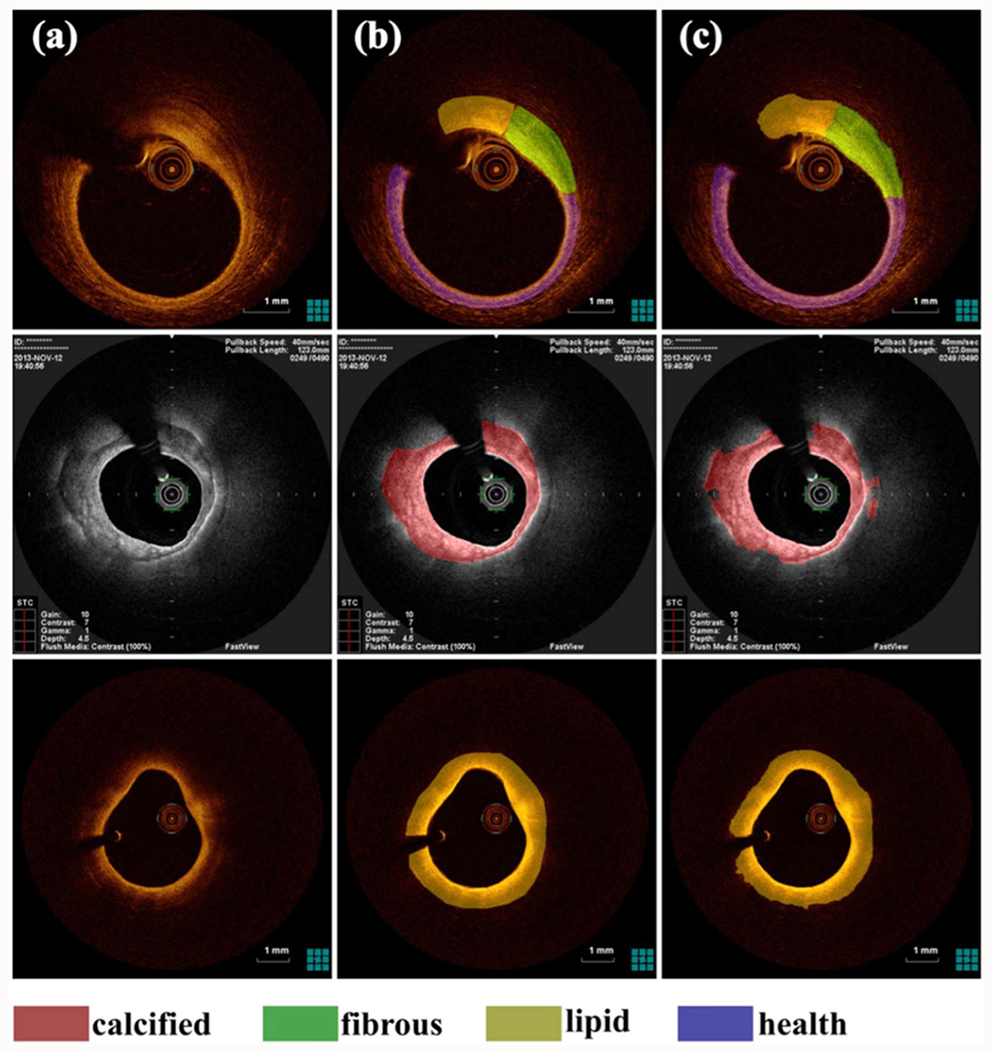
Successful pixel-wise classification examples from certain datasets. column-(a) IVOCT image, column-(b) ground-truth, and column-(c) illustrates the segmentation results obtained with our proposed deep learning neural network. The annotation colors for each tissue is denoted at the bottom of the resulting plane.

Some studies have suggested that multi-step approaches could yield better segmentation results compared to one-step methods. For instance, Lee from our group [58] proposed a two-step deep learning method for characterizing coronary calcified plaque. The first step used a shallow 3D CNN for major lesion detection, followed by SegNet for detailed plaque segmentation. Compared to the standard one-step approach, this two-step approach significantly improved sensitivity (from 77.5% to 86.2%), precision (from 73.5% to 75.8%), and F1 score (from 0.749 to 0.781). Similarly, a three-step framework for plaque segmentation in IVOCT images was proposed [59], using U-Net for lumen segmentation and a self-attention ResNet for image classification. Although the classification performance was exceptional (100% sensitivity for lipid and calcification), the segmentation step showed lower Dice coefficients of 60.5% and 71.8% for lipid and clacification, respectively. Another two-step approach for plaque characterization [60] first segmented the outer border of the ROI using a level-set method to isolate the visible superficial layer. Cropped square patches from the ROI were then used as input for a dense-block-SegNet (DBSegNet) for detailed segmentation. This approach achieved sensitivities of 91.8% for both calcified and lipid plaques and 92.8% for fibrous plaques (Fig. 3).

There have also been attempts to develop integrated plaque characterization software using deep learning. For example, a deep convolutional network with a U-shaped encoder-decoder architecture was developed [61], utilizing pseudo-3D input from consecutive IVOCT cross-sections. This model, trained on a dataset comprising 11673 images from 509 pullbacks, achieved Dice coefficients of 0.906 for fibrous plaques, 0.848 for calcified plaques, and 0.772 for lipid plaques, demonstrating excellent agreement in plaque burden quantification (*R^2^* = 0.98). The model was integrated into the commercial software OctPlus, developed by Shanghai Pulse Medical Technology, Inc. Building on extensive experience with IVOCT imaging, our group [62] introduced the Optical Coherence TOmography PlaqUe and Stent (OCTOPUS), a comprehensive analysis software for coronary plaques and stents in IVOCT images. The software quantifies various aspects, including stent deployment characteristics, strut level analysis, calcium angle, and calcium thickness. Incorporating deep learning for plaque segmentation and machine learning for stent strut identification, OCTOPUS reduced manual editing time by approximately 80%, with only 3.8% of plaque pixels requiring manual adjustments.

To effectively train deep learning models, it is important to select images that represent various types of plaques and their characteristics. Our group explored methods to reduce the number of images requiring detailed annotation, thereby minimizing the effort needed for manual labeling [63]. We tested different percentages (10–100%) of training images derived from two approaches: equally spaced image subsampling and deep-learning clustering. The first approach involved selecting images at fixed intervals from the volume, while the deep-learning clustering method used an autoencoder to create a deep feature space representation, followed by k-medoids clustering, with the cluster medians used for training. For a given sampling ratio, the deep-learning clustering method performed as well as or better than the equally spaced sampling approach and the method trained using all labeled data. Our results highlight strategies to reduce the significant effort needed for annotating images, especially in applications with high image variability.

In addition, several additional patch-wise classification studies on IVOCT images have contributed to advancements in plaque characterization. For example, one study [38] identified and characterized pathological lesions in IVOCT images, classifying them as calcification or fibrosis. They extracted features using three CNN networks (AlexNet, VGG-19, and Inceptionv3) and employed random forest classifiers with majority voting for final classification, achieving sensitivities/specificities of 0.84/0.95 and 0.94/0.96, respectively. Another study [64] proposed a semi-automated algorithm for plaque identification, relying on texture features from manually selected ROIs. They focused on classifying atherosclerotic plaques (calcium, lipid, and fibrous tissue), achieving an average accuracy of 90.3% in plaque characterization. Additionally, a study [65] detected plaque erosion using a deep learning approach that combined Mask R-CNN for initial mask identification with SVM for classification. To label 3D connected regions, they employed 26-connectivity neighborhood system [66]. Their method showed a sensitivity of 0.800, a precision of 0.734, and an AUC of 0.707.

##### C. Segmentation of Microscopic Elements in Coronary Plaques

With its near-histologic resolution and optical contrast, IVOCT enables the visualization of microscopic elements in coronary plaques associated with vulnerability, such as TCFA, microchannels, cholesterol crystals, and macrophage infiltration. In IVOCT images, TCFA is defined as a thin fibrous cap with a thickness of less than 65 *μm* overlying a large lipid pool [67]. Microchannels appear as signal-free tubuloluminal structures with no connection to the vessel lumen, visible in more than three consecutive IVOCT frames [20]. Cholesterol crystals are identified as linear regions of high intensity [68], and macrophage infiltrations show up as punctate, signal-rich spots that exceed the background noise of the image [68]. However, the identification of these microscopic plaques has been the focus of only a few research groups, primarily relying on supervised deep learning. Supplementary Table S1 provides a summary of the relevant segmentation studies.

Building on a 2018 study [41], one research team [38] used features from AlexNet, VGG-19, and Inceptionv3 CNNs for macrophage detection in IVOCT images, employing random forest classifiers and majority voting for final classification. They achieved a specificity of 0.97, sensitivity of 0.89, and accuracy of 0.92. Another study [69] developed a simple image processing method for automated detection of macrophage infiltration in coronary plaques. By calculating normalized-intensity standard deviation values and using an optimized threshold, they attained around 88% sensitivity and specificity from postmortem coronary segments.

**Fig. 4.**
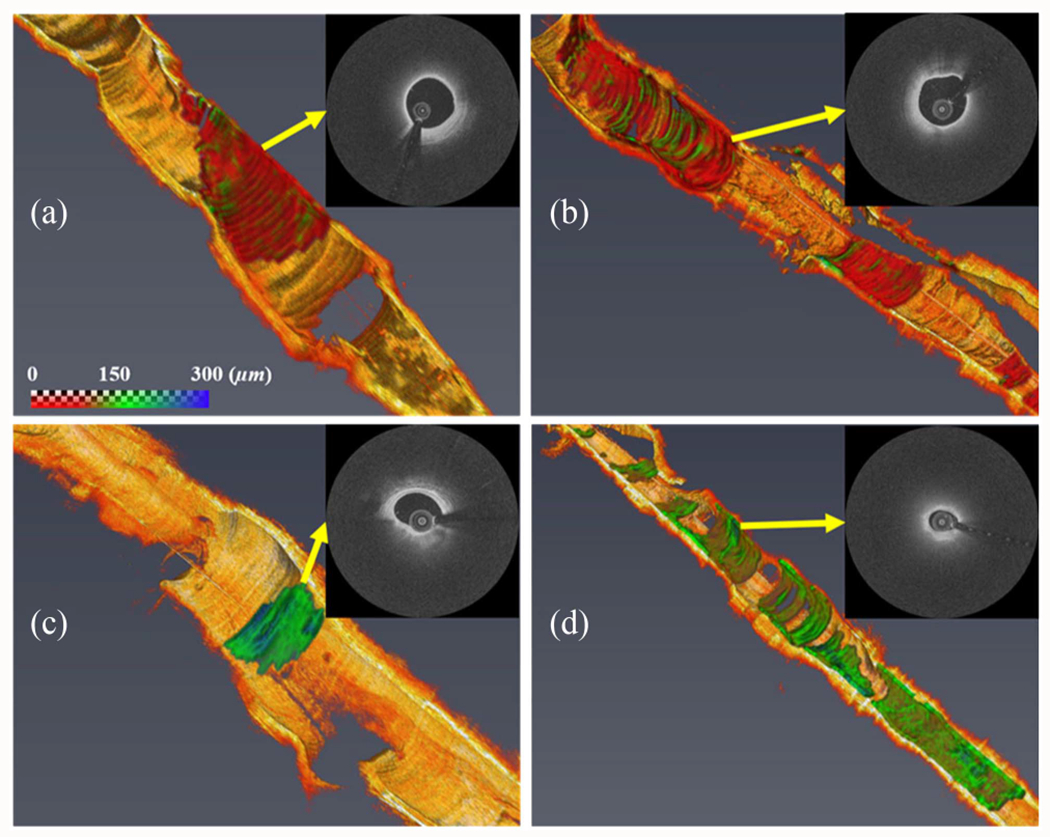
Three-dimensional (3D) visualizations of fibrous cap thickness on the representative IVOCT pullbacks, including: (a) short lesion with TCFA, (b) long lesion with TCFA, (c) short lesion without TCFA, and (d) long lesion without TCFA. The reader can zoom in each artery to see variations of fibrous cap thickness. (a) Although the lesion length was not too long (< 7 mm), the average fibrous cap thickness was less than 65 *μ*m across the lesion indicating that the lesion is prone to rupture. (b) There were two lipidous lesions having 15 mm (left) and 5 mm (right) lengths. Both lesions were heavily lipidic with a mean cap thickness of < 65 *μ*m. The artery was much more prone to rupture than (a). (c) The lesion was stable, since the length was short (< 3 mm) and the fibrous cap thickness was always greater than 150 *μ*m. (d) Although the fibrous cap thickness was always over 80 *μ*m across the lesion, the lesion length was very long (> 30 mm). There were several spots approaching toward the vulnerable plaque than (c). The color map visualizes the fibrous cap in the range of 0 to 300 *μ*m. The yellow arrows indicate representative IVOCT frames of each rendering. Our method provides comprehensive fibrous cap map in the entire IVOCT pullback, so clinicians can make appropriate treatment decisions.

Automated fibrous cap segmentation has been explored in several studies using traditional machin learning [70], semiautomated deep learning [71], and fully automated end-to-end deep learning approaches [61], [72]. Using traditional machine learning features (e.g., local binary patterns and gray level co-occurrence matrices) combined with SVM, lipid plaque borders and fibrous cap thickness were characterized [70]. The SVM model showed 89.6% sensitivity and 78.6% specificity for lipid identification, with an 8.6% error in cap thickness measurement compared to manual assessment. Additionally, a semi-automated deep learning method [71] was developed to detect lipidous plaque and assess fibrous cap thickness in IVOCT images (Fig. 4). The DeepLab-v3 plus deep learning model was used to determine the extent of lipidous plaque. Following lipid detection, the outer border of the fibrous cap was determined using a dynamic programming algorithm. This method exhibited excellent discriminability for lipid plaque, achieving a sensitivity of 85.8% and an A-line Dice coefficient of 0.837. Automated cap thickness measurement required significant modifications in only 5.5% of frames. For fully automated end-to-end segmentation, Chu et al. [61] employed a U-shaped encoder-decoder deep learning architecture with pseudo-3D input from stacked IVOCT frames. They addressed class imbalance using a hybrid loss function combining cross-entropy and focal Tversky losses, achieving moderate precision, recall, and Dice coefficients for different plaque components. In a recent study [72], a fully automated deep learning method was specifically designed for the segmentation of fibrous caps. This method was developed and externally validated using substantial IVOCT datasets, comprising more than 32,000 images from 227 pullbacks. It provided highly reliable segmentation results during both five-fold cross-validation and external validation, achieving Dice coefficients of 0.846 and 0.816, respectively. Moreover, the discrepancy in fibrous cap thickness measurements between the manual and automated methods was minimal (~3.0 *μ*m).

Our group [73] focused on identifying microchannels in IVOCT images using deep learning. We employed the DeepLab v3+ model for initial segmentation, followed by a CNN with three convolutional layers, two maximum pooling layers, and a fully connected layer to classify each candidate as a microchannel or non-microchannel. Data augmentation was applied to both raw polar IVOCT images and microchannel candidates. The classification network achieved high accuracy with a sensitivity of 99.5% and specificity of 98.8%. This step increased the Dice coefficient for microchannel segmentation from 0.71 to 0.73.

##### D. Non-Tissue Structures

Some studies have been potentially helpful in aiding comprehensive vessel analysis in IVOCT images, including side branch and catheter detection (Table S1). One such study [74] automated side branch ostium detection by first segmenting the lumen boundary using dynamic programming and then identifying side branch ostium points. This method introduced a novel curvature definition through global view angle calculations, achieving a sensitivity of 82.8%, specificity of 98.7%, and precision of 86.8%. Another group of studies [54], [56], [58], [71], [73] effectively removed guidewire and shadow regions in IVOCT images using an accumulated intensity map and dynamic programming. Additionally, a U-shaped encoder-decoder architecture was employed to detect non-tissue structures, such as the guidewire and side branches, achieving high precision, sensitivity, and Dice coefficients for both structures [61]. Another approach [75] proposed an automatic side branch detection method that utilizes adjacent frame correlation. This method consists of three main steps: computing the distance in a single frame, calculating the width gradient of the candidate region in the adjacent frame, and conducting a quantitative analysis of the side branch. By employing 19 IVOCT pullback runs, they achieved an F1 score of 87.3%, compared to manual segmentation.

#### IV. Stent Analysis

Coronary stents are frequently used in PCI to treat significant obstructive lesions. Numerous clinical studies have shown that stent strut distribution in the vessel wall can affect the outcome of PCI [76], [77], [78]. In particular, some studies have suggested that inadequate stent strut coverage and apposition may be associated with an increased risk of short- and long-term adverse outcomes [79], [80], [81], [82]. Several stent trials have used strut coverage, assessed by IVOCT, as their primary end point [83], [84], [85], [86], [87]. Accurate quantitative analysis requires precise segmentation that can detect lumen contours and stent struts. Consequently, various machine learning [88], [89] and supervised deep learning [90], [91], [92] approaches for stent analysis have been reported (Table S1).

**Fig. 5.**
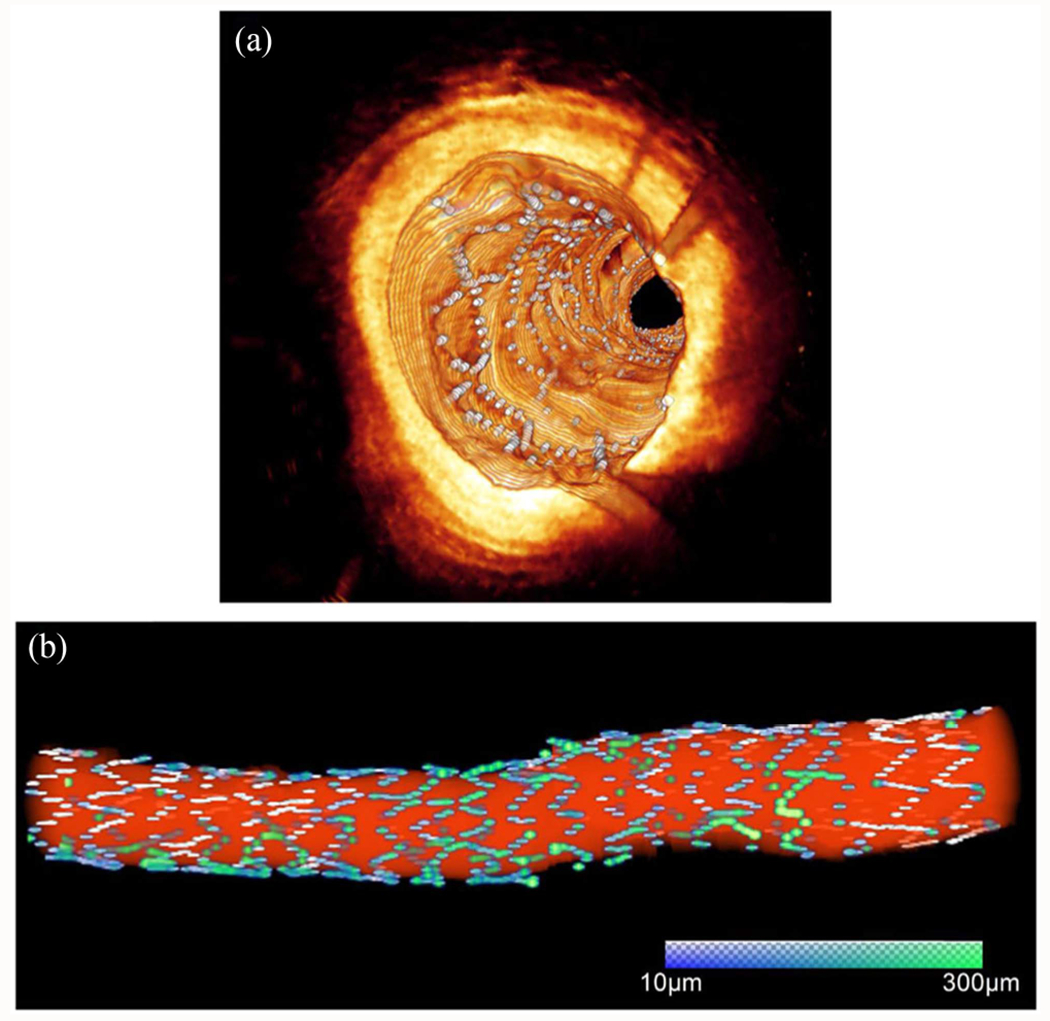
3D visualization of stent. (a). Blood vessel wall is rendered in gold and stent is in grey. (b). Vessel wall is removed. Lumen is rendered in red. Uncovered struts are in white and struts with different tissue coverage thickness are color coded in blue and green.

In a landmark machine learning study, Wang et al. [88] proposed a novel method for stent detection in IVOCT images using a Bayesian network and graph search. Initially, they computed the probability of stent strut appearance using a Bayesian network and analyzed stent wire continuity from adjacent frames in an *en face* view. Subsequently, they localized the depths of all the stent struts in a pullback using a graph cut algorithm. Utilizing more than 8000 clinical images form 103 pullbacks, their method achieved a recall of 0.91 and a precision of 0.84. Another study [89] developed highly-automated algorithms for stent strut detection and classification in IVOCT images. Building on their group’s prior algorithms [93], they first detected stent struts, then trained a SVM model using 21 features to classify each strut as covered or uncovered. This approach demonstrated excellent classification performance, with a sensitivity of 94% and specificity of 90% (Fig. 5). They also improved tissue coverage thickness measurement, surpassing the accuracy of commercial products.

For deep learning approaches, Jiang et al. [90] proposed two automatic methods for detecting metallic stent struts using YOLOv3 and R-FCN networks. To enhance performance, they implemented data augmentation by rotating images and adjusting anchor box sizes. Both methods achieved over 95% in precision and sensitivity, with R-FCN outperforming YOLOv3 across all metrics. Additionally, a U-shape-like deep learning architecture was employed for stent strut detection [91]. Using pseudo-3D images to aggregate information from adjacent frames, this method was independently tested on an extensive dataset of 21,363 images from 170 IVOCT pullbacks, achieving outstanding segmentation (Dice: 0.907) and detection results (precision: 0.943, sensitivity: 0.940). Most recently, another study [92] utilized a U-Net model combined with MobileNetV2 and DenseNet121 to segment two major clinical stent types, including metal stents and bioresorbable vascular scaffolds (BVS). To address the imbalance of background and stent strut pixels, they cropped each image into multiple sub-images for network training, resulting in sub-images containing stent struts. Their method demonstrated a Dice coefficient of 0.86 for BVS segmentation and a precision/sensitivity of 0.92/0.92 for metal stent segmentation.

Lu from our group [94] introduced the OCT Image Visualization and Analysis Toolkit for Stent (OCTivat-Stent), a toolkit for automated IVOCT pullback analysis. This software automates the detection of the guidewire, lumen boundary, and stent struts, and quantifies tissue coverage and stent contour. It also calculates strut-level tissue thickness, coverage, and malapposition area, leading to a 30% reduction in inter-observer variability.

#### V. OCT-Derived Fractional Flow Reserve (FFR)

**Fig. 6.**
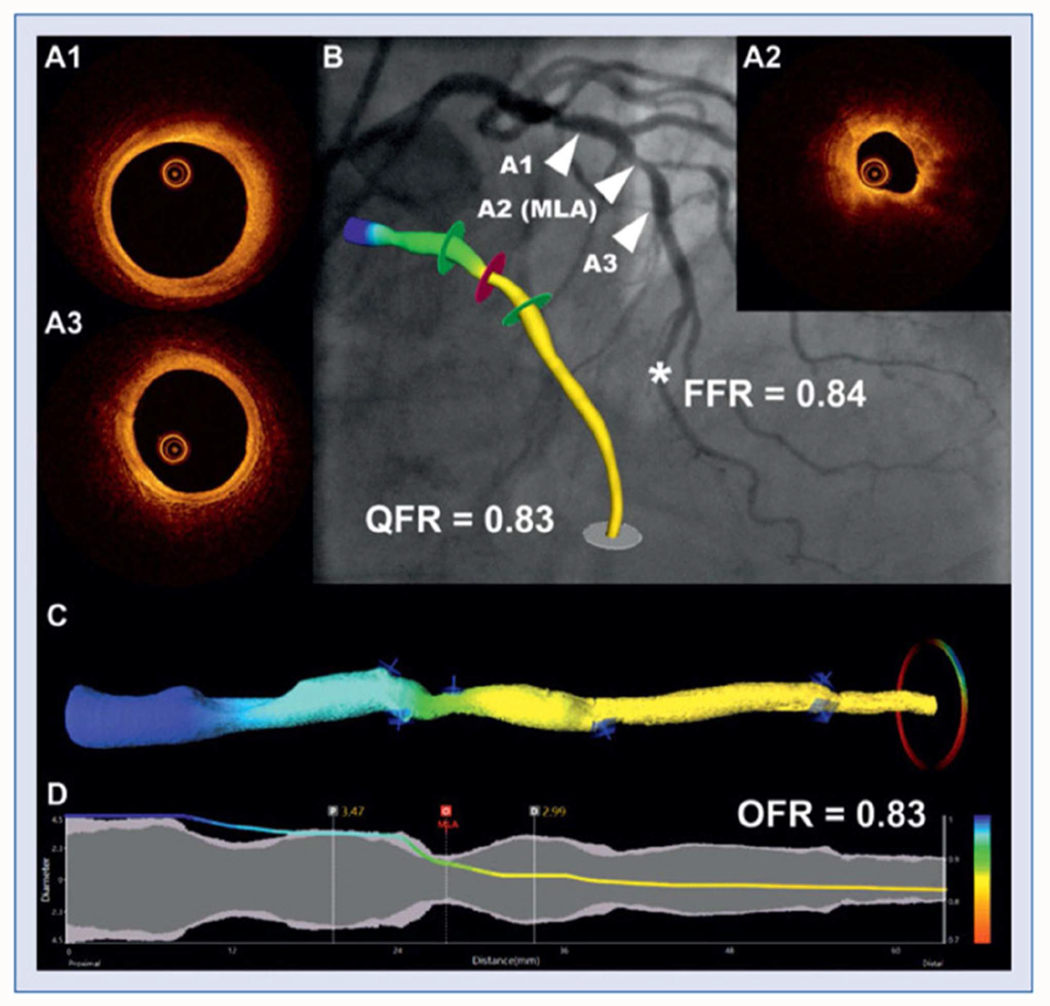
Computation of optical flow ratio (OFR) and quantitative flow ratio (QFR) in an intermediate stenosis in the left anterior descending artery. Cross-sections A1–A3 correspond to the angiography positions showed in panel B (arrow heads), with A2 as minimal lumen area (MLA). Fractional flow reserve (FFR) measured at a distal position (b,*) was 0.84. The computed QFR (b) and optical flow ratio (OFR) (c) along the vessel are color-coded and superimposed on a three-dimensional reconstruction, both with a value of 0.83 at the marked point (*). The software renders a virtual pressure pullback within the coronary artery (d) for optimal co-registration between pressure-drop and anatomy.

FFR plays a crucial role during PCI by providing a physiological measurement of the severity of coronary artery stenosis through the pressure gradient across a lesion. This measurement aids in the decision-making process for stenting and optimizes patient outcomes [95]. Since IVOCT enables precise reconstruction of vessel dimensions, several studies have developed IVOCT-derived FFR using computational fluid dynamics (CFD) [96], [97], [98], [99] (Fig. 6) and machine learning [100] methods. Supplementary Table S1 summarizes studies focusing on IVOCT-based FFR measurements.

Using CFD, Tian et al. [96] introduced the novel software package, enabling automated computation of OCT-based FFR (OFR) from IVOCT pullbacks. The software, primarily based on hyperaemic volumetric flow rate at the inlet boundary, has undergone validation in multiple studies [97], [98], [99]. One such study [97] involved computational analysis in 125 vessels from 118 patients, where OFR demonstrated an accuracy of 90%, sensitivity of 87%, and specificity of 92% in identifying wire-based FFR ≤0.8. The average analysis time for OFR was 55±23 seconds per IVOCT pullback, with minimal intra- and inter-observer variability (0.00±0.02 and 0.00±0.03, respectively), highlighting its high clinical applicability. However, limitations exist in clinical practice due to constraints in OCT coronary geometry, such as the absence of side branch geometry and the prolonged time for 3D reconstruction and CFD simulation.

Addressing these limitations, another study [100] developed a machine learning model to predict FFR using IVOCT images. They selected 36 features based on expert opinion and literature review, including epidemiological data, medical history, and plaque characteristics. The random forest model demonstrated good correlation with wire based FFR (*R* = 0.853, *p*<0.001) and high accuracy in classifying FFR ≤0.8, with sensitivity, specificity, and accuracy in the testing group being 100%, 92.9%, and 95.2%, respectively.

#### VI. IVOCT Pullback Registration

IVOCT-IVOCT pullback registration is essential for clinical studies on stenting outcomes, major adverse cardiovascular events (MACE), neoatherosclerosis, and CAV (refer to Section X for details). Despite its importance, there are only a few studies on IVOCT pullback registration (Table S1). One notable study [101] developed an automated method for registering pre- and post-stenting IVOCT pullbacks. The process began with segmenting calcifications in raw polar IVOCT images using deep learning. They then generated 1D representations of calcium thickness along the pullback and registered the pullbacks by calculating the cross-correlations between pairs of 1D graphs. This approach demonstrated that each frame matched its corresponding image more accurately than adjacent frames, indicating a registration accuracy within a 1-frame interval (±200 *μm*).

#### VII. Computer Simulations Using IVOCT-Image-Derived Finite Element Analysis and Computational Fluid Dynamics

Computer simulations, including finite element analysis (FEA) and CFD, have become powerful tools for studying the complex interactions between stents and arteries, as well as the hemodynamic alterations following stenting procedures. Initially, computer simulations focused on uncovering the mechanisms of stenting in complex lesions using stylized models. However, with advancements in imaging technologies, image processing software, and computing capabilities, patient-specific models have been developed from IVUS or IVOCT images. These developments allow for more accurate predictions of stenting outcomes and pave the way for more advanced stenting planning tools.

##### A. FEA for Stent-Artery Interaction

**Fig. 7.**
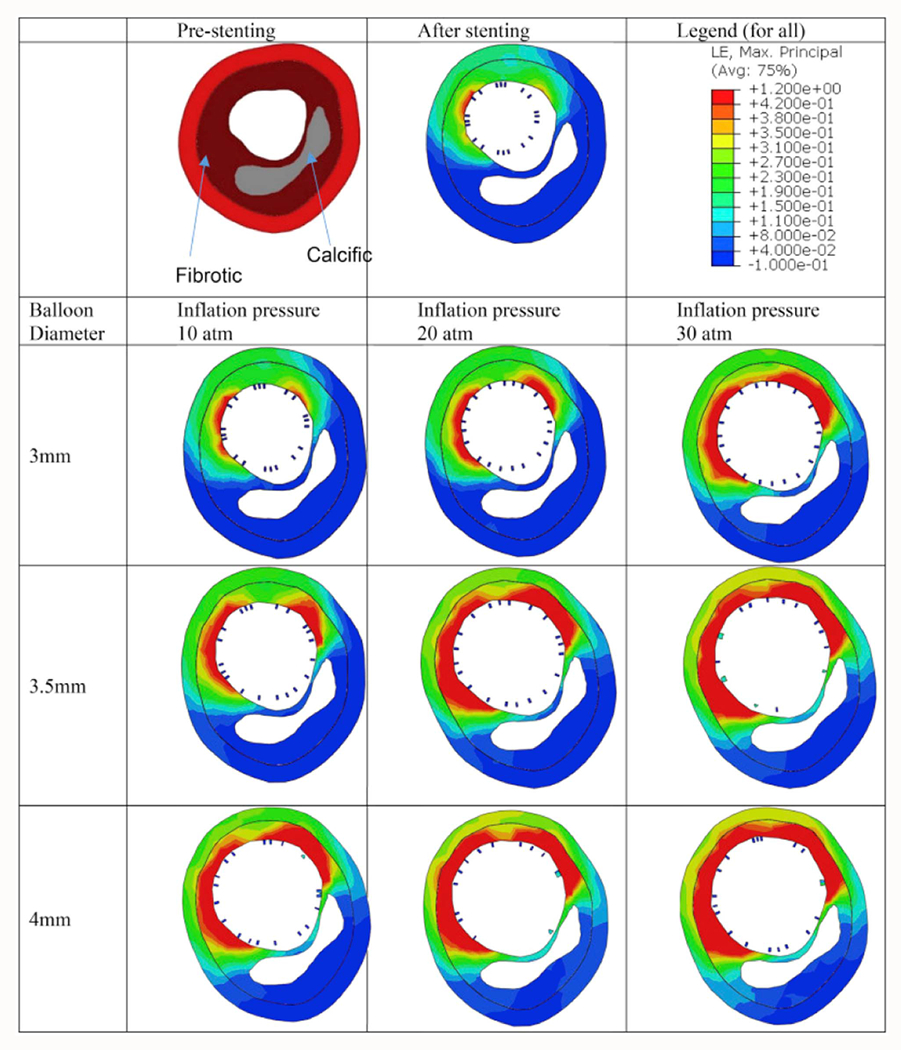
Strain distribution in the fibrotic tissue and calcification at a representative cross section Z = 10 mm.

Patient-specific artery models reconstructed from IVOCT images have been utilized to evaluate stenting procedures in complex lesions. For example, one study [102] developed such a model to study stent overlap in long lesions. The results indicated that stent overlap leads to greater lumen gain but also poses a higher risk of tissue damage and restenosis. Consequently, they recommended using multiple stents with minimal overlaps when a single stent is not feasible. In another context, heavily calcified coronary arteries were assessed [103] to understand how calcification attributes influence stent expansion using a patient-specific model based on IVOCT images. They quantified cross-sectional calcification attributes (e.g., angle, maximum thickness, and area) at various longitudinal locations, revealing that stent expansion is correlated with the calcification angle and area. Additionally, another study [104] examined the impact of different post-dilation parameters on the effectiveness of enhancing stent expansion in heavily calcified coronary arteries. FE simulations were conducted alongside ex vivo experiments, which involved post-dilations using balloons of increasing diameters and inflation pressures for each diameter (Fig. 7). The FE simulations aligned well with the ex vivo experiments, showing similarities in the stented lumen area and stent malapposition. The results suggested that using a balloon at a higher inflation pressure could safely improve the lumen area. Furthermore, the stress distribution across the vessel wall indicated that increased fibrosis stretch after each post-dilation enhances the stented lumen area. A large calcification angle was found to reduce the vessel’s stretchability, leading to stent under-expansion. The initial lumen area and the circumferential length of fibrosis, indicating the amount of stretchable tissue, were key factors in determining the potential for stent expansion and the risk of vessel rupture. These could serve as indices for optimal stenting. In summary, the development of patient-specific FE models from IVOCT images marks significant progress toward evaluating and predicting stenting outcomes in clinical scenarios.

##### B. CFD Analysis for Stent-Artery Interaction

By integrating FEA and CFD simulations, one study [105] assessed the hemodynamic alternations following stenting and post-dilation in a heavily calcified coronary artery. The fluid domain was reconstructed based on the FE simulation results, and CFD simulations were then conducted to quantify the hemodynamic alternations. Pulsatile blood flow was simulated using a Windkessel-type boundary condition. The analysis of the instantaneous wave-free ratio (iFR) demonstrated the efficacy of the post-dilation procedures. Another study [106] investigated the influence of side branches, located upstream and downstream of the stenosis lesion, on FFR evaluation using CFD simulations. The results indicated that the upstream side branch had a minimal effect on FFR evaluation, whereas a downstream branch with a larger diameter (greater than one-third of the main vessel’s diameter) resulted in a lower FFR value. These findings were validated by clinical measurements.

#### VIII. IVOCT Plaque Characteristics Used to Refine CCTA Analyses

IVOCT imaging has been used to identify the presence and characteristics of coronary atherosclerosis. However, its invasive nature limits its applicability, particularly in patients early in the disease process. In contrast, coronary computed tomography angiography (CCTA) is a non-invasive imaging modality that enables assessment of both luminal stenosis and atherosclerotic plaque morphology throughout the entire coronary tree [107], [108], [109]. While clinical interpretation of CCTA has traditionally focused on luminal stenosis, emerging evidence suggests that analyzing various plaque and vessel wall features may further enhance risk stratification [110], [111], [112], [113], [114], [115]. These findings support the further development of quantitative CCTA assessments. A few studies have even begun exploring the prediction of IVOCT-defined plaque characteristics using CCTA image analysis (Table S1).

For example, one study [116] explored whether conventional plaque assessment and radiomics in CCTA could predict high-risk plaques as determined by IVOCT. They analyzed seven conventional plaque features and calculated 935 radiomic parameters, including first-order statistics and gray level matrices. Using stratified cross-validation with 1,000 repeats to minimize overfitting, they found that the fractal box counting dimension of high attenuation voxels was most accurate in identifying IVOCT-defined TCFA (AUC: 0.80, CI: 0.72-0.88). Low attenuation plaque was the most effective conventional metric (AUC: 0.66, CI: 0.58-0.73). Another investigation [117] examined the association between PCAT radiomics from CCTA images and IVOCT-defined vulnerable-plaque characteristics (i.e., microchannel and TCFA). They extracted 1356 radiomic features from the lesion and the entire vessel, using stratified cross-validation with 1,000 repeats to evaluate the predictability. Both lesion-based and vessel-based methods performed similarly in identifying TCFA and microchannel, with comparable accuracy (88% for TCFA and 91% for microchannel). Most recently, a study [118] compared different plaque types between CCTA and IVOCT. After co-registering CCTA and IVOCT scans using distance to reference points, they found CCTA’s diagnostic performance to be excellent in detecting any IVOCT-identified plaque, with sensitivity, specificity, and accuracy of 92%, 98%, and 93%, respectively. This suggests CCTA’s effectiveness in identifying coronary plaques, except for sub-millimeter sizes indicative of early atherosclerosis.

#### IX. Clinical Outcomes Research Using Quantitative IVOCT Assessments

IVOCT has been utilized in numerous clinical studies due to its ability to provide a comprehensive assessment of both macroscopic and microscopic coronary plaques, which are predictive of future risk. In this section, we have categorized these studies into five groups: stenting outcomes, MACE, neo-atherosclerosis, target vessel failure (TVF), and CAV applications (Table S1).

##### A. Stenting Outcome Prediction

**Fig. 8.**
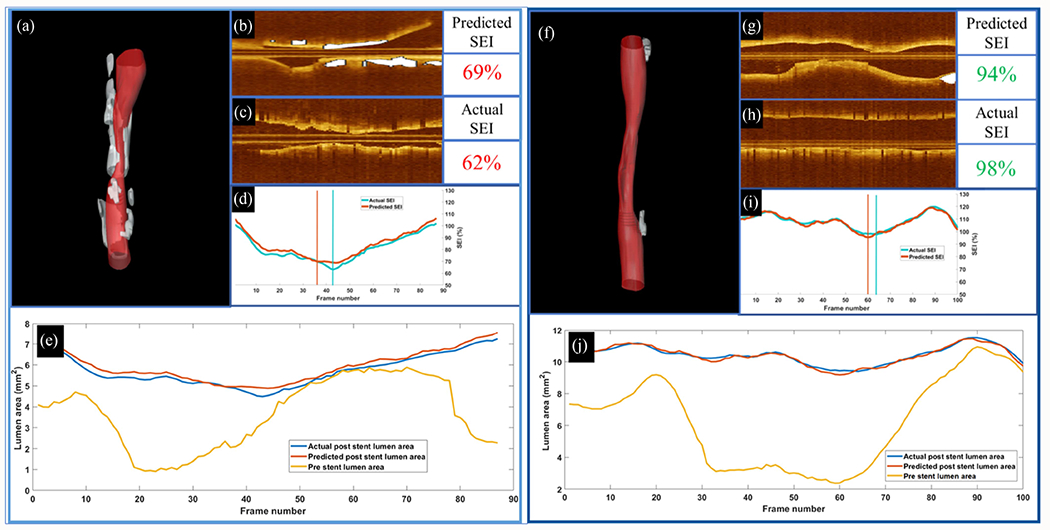
Predicted stent area in cases with different calcifications severity. Predicted stent area in a case of under-expansion in a heavily calcified lesion (left panel (a)–(e) and a case with a well-expanded stent in a vessel with relatively little calcification (right panel (f)–(j)). (a)–(f) Three-dimensional rendering with calcifications in white, (b)–(g) longitudinal view before stenting with calcifications in white, (c)–(h) longitudinal view after stenting, and (d)–(i) predicted (orange) and actual (green) SEI following stenting. Our method predicted an SEI of 69%, which is close to the actual value of 62%, in which both values were indicative of under-expansion. The vertical bars in (d)–(i) show the locations corresponding to the minimum SEI values. The closeness of their location further suggests the predictive value of the regression model. The predicted and actual SEIs were 94 and 96%, respectively, and their locations were very close together. (e) and (j) show the effect of calcifications on stent expansion. Predicted and actual lumen areas after stenting are in blue and red, respectively. The orange curve represents the pre-stent lumen area for the registered pullback. In (e), areas were not enhanced after stenting because of the presence of calcifications (frames 50–70). (j) Is associated with a well-expanded stent (frames 25–80).

In the landmark study, Fujino et al. [28] introduced a novel IVOCT calcium scoring system for predicting stent expansion index (SEI). Utilizing multivariable linear regression, they developed a scoring system where points were assigned based on calcium angle (>180° = 2 points), thickness (>0.5 mm = 1 point), and length (>5 mm = 1 point). Their OCT calcium score demonstrated better predictive accuracy for stent expansion <70% compared to coronary angiogram (AUC: 0.86 vs. 0.84, *p* = 0.79). Building on this approach, another study [29] created an advanced machine learning model to predict vessel expansion success as measured by SEI. They first segmented the lumen and calcifications in pre-stent IVOCT images using deep learning and extracted 39 features, including 18 lumen and 21 calcification features. Using LASSO for feature selection, their Gaussian regression model achieved outstanding performance (root-mean-square-error = 0.04 ± 0.02 mm*^2^*, *R* = 0.94 ± 0.04, *p*<0.0001) when combining lumen and calcification features over 31 frames (Fig. 8). This method significantly surpassed the previous state-of-the-art method [28].

##### B. MACE Prediction

One investigation [119] examined the link between macrophage infiltration detected by IVOCT and MACE. They found a significantly higher MACE rate in patients with plaque erosion and macrophage infiltration (21.6%) compared to those without (5.9%, *p* = 0.008). Multivariable Cox regression identified plaque erosion with macrophage infiltration [hazard ratio (HR) = 2.95, 95% CI: 1.09-8.02, *p* = 0.034] as independent MACE predictor. Another study [120] evaluated the prognostic significance of plaque characteristics and residual syntax score (rSS) in predicting MACE. They discovered that the presence of TCFA in culprit lesions and higher rSS levels were significantly linked to MACE occurrence. A combination of rSS and TCFA yielded the highest prediction accuracy (AUC: 0.816, 95% CI: 0.765-0.860). Additionally, a study [82] analyzed post-stenting IVOCT images to identify predictors of device-oriented clinical endpoints (DoCE) and major safety events (MSE). Key findings included that a smaller minimal stent area and total malapposition volume ≥7.0 mm*^3^* were independent predictors of DoCE and MSE, respectively. Additionally, they noted that a total malapposition volume ≥7.0 mm*^3^* post-stent was associated with an increased incidence of late malapposition and uncovered struts.

##### C. Neo-Atherosclerosis Prediction

Our group [121] focused on the predictive capability of IVOCT-defined plaque characteristics for in-stent neoatherosclerosis. The study analyzed images before and 18 months post-stent implantation, including 180 lesions from 90 patients. We evaluated 17 plaque features from baseline IVOCT images, such as lesion length, lumen area, calcium angle and thickness, and fibrous cap characteristics (thickness, surface area, and burden). Multivariate logistic regression analysis revealed that a larger fibrous cap surface area was strongly associated with the development of neo-atherosclerosis (odds ratio 1.38, 95% CI: 1.05-1.80, *p*<0.05).

##### D. TVF Prediction

Another study [122] investigated the prevalence of neoatherosclerosis after PCI and its impact on long-term outcomes using IVOCT imaging. The findings linked renal insufficiency and poor lipid to lipidic neo-atherosclerosis, and severe renal insufficiency and female sex to calcified neo-atherosclerosis. Female sex (HR = 2.05, 95% CI: 1.36-3.09, *p*<0.001) and lipidic neo-atherosclerosis (HR = 1.56, 95% CI: 1.06-2.30, *p* = 0.03) were associated with an increased incidence of TVF. Another study [123] analyzed clinical and procedural variables to predict TVF using ultrathin-strut drug-eluting stents. Key predictors identified included age, ST elevation myocardial infarction, reduced left ventricular ejection fraction, diabetes, and renal dysfunction. They also discovered that the use of intracoronary imaging, specifically IVOCT, significantly reduced TVF risk. In another study [124], clinical outcomes between IVOCT-guided and IVUS-guided PCI were compared in a large cohort of 2008 patients. Although lacking sufficient statistical power, their findings suggested that IVOCT-guided PCI was not inferior to IVUS-guided PCI.

##### E. CAV Prediction

Numerous studies have performed quantitative assessments of IVOCT pullbacks for the early diagnosis of CAV [30], [125], [126], [127], [128], [129], [130], [131], [132], [133], [134], [135], [136]. For instance, one study [125] analyzed 50 baseline and follow-up IVOCT pullbacks post-heart transplant using a 3D LOGISMO graph-based approach. They observed significant changes in mean luminal area and intimal thickness within the first-year post-transplant, particularly in the proximal parts of the vessels. Another investigation [126] characterized quantitative and qualitative IVOCT measurements in 82 patients to determine indicators of early CAV progression. The study observed significant increases in median mean intimal thickness, intimal volume, and percentage intimal volume from baseline to follow-up, along with a decrease in lumen volume. Another study [127] investigated whether CAV causes similar vascular remodeling in major coronary arteries. They analyzed IVOCT pullbacks at 1-mm intervals using QCU-CMS software (Medis Medical Imaging, The Netherlands) and measured luminal area, intimal area, intimal thickness, and medial area. Measurements in the LAD were identified as stronger predictors of CAV progression, suggesting that LAD measurements are superior for CAV prediction.

There have also been interesting studies analyzing pediatric heart transplant recipients using quantitative IVOCT assessment. Cote et al. [128] investigated the relationship between intimal thickening and ventricular function in 17 pediatric heart transplant recipients, finding significant correlations with longitudinal diastolic strain rate and stroke volume index. Another comparison [129] of intimal thickness and intima/media cross-sectional area ratios in pediatric recipients observed greater thickness in cases with rejection or concurrent CAV, especially in patients not treated with statins. Both studies demonstrated that IVOCT can provide insights into coronary vascular changes in pediatric transplant recipients, which are not detectable by angiography. Further research [130] compared quantitative IVOCT measurements between pediatric and adult heart transplant patients at two post-transplant intervals. They observed that relative intimal hyperplasia peaked early in children and was significantly more pronounced in the pediatric cohort than in the adult cohort. These results imply that the prevalence of IVOCT findings may vary according to age- and time-dependent differences.

#### X. Future Directions

With its unprecedented resolution, IVOCT provides new research opportunities. Assessing detailed plaque structures (e.g., TCFA and microchannels) at the microscopic level will enhance our understanding of the pathobiology of individual plaque structures. Integrating known risky plaque characteristics with new microscopic biomarkers could provide deeper insights into the mechanism of coronary atherosclerosis. While serial IVOCT imaging is valuable for clinical studies, accurate and automated registration of baseline and follow-up pullbacks is essential. Using AI techniques to correlate IVOCT findings with patient data may help identify those at high risk for future adverse events, such as MACE and TVF.

While several small studies have indicated a potential role for IVOCT in enhancing PCI, there is no substantial evidence confirming its superiority over IVUS or angiography in improving clinical outcomes. However, the automatic interpretation of images, including those of TCFA and plaque erosion, could facilitate the widespread clinical application of IVOCT. Since IVOCT imaging enables quantitative assessment of calcification and lipid extent, it can help select the best treatment plan and adjust the landing zone and stent length to avoid placing rupture-prone plaques (e.g., TCFA) at the edge of the stent. Additionally, automated IVOCT analysis can accurately predict post-stenting outcomes (e.g., SEI) prior to stenting, as reported by [28], [29]. For instance, if the target vessel is at risk of under-expansion, the interventional cardiologist can opt for a plaque modification method (e.g., atherectomy). Furthermore, automated analysis has the potential to identify non-culprit lesions that should be treated despite the lack of significant obstruction. Consequently, further research is needed to evaluate the benefits of quantitative IVOCT-based management in patients with ACS.

Another promising technique for enhancing the clinical value of IVOCT imaging is the integration of near-infrared fluorescence (NIRF) to target specific coronary plaques, such as lipid-rich plaque [137], [138], [139], [140]. This dual-modality imaging method allows for the simultaneous acquisition of detailed structural and molecular information from atherosclerotic plaques. The use of targeted contrast agents, such as porphyrin lipid nanoparticles, enables NIRF to highlight specific biological markers within plaques, enhancing anatomical detail. Combining this technique with deep learning can further enhance clinical and research potential. Deep learning algorithms can analyze the extensive data generated by NIRF-OCT imaging to identify patterns and predict outcomes with high accuracy. These algorithms can facilitate the automated detection and characterization of high-risk plaques, potentially improving diagnosis and treatment planning. By integrating deep learning, the NIRF-OCT technique can deliver real-time, precise assessments of plaque composition and stability, ultimately leading to better-informed clinical decisions and improved patient outcomes.

Enhanced and automated plaque characterization in IVOCT could pave the way for personalized treatments by enabling clinicians to make more informed decisions. Over the past decade, the development of preventive and cardioprotective therapies, such as P2Y12 antagonists, direct oral anticoagulants, PCSK9 inhibitors, icosapent ethyl, and GLP-1 agonists, has highlighted the need for personalized medicine. This approach ensures that each patient receives the most appropriate treatment in a cost-effective manner. By automating the identification of high-risk vessels, we can better guide intensive therapies in clinical settings and select suitable cohorts for testing new therapies. Accurately determining high-risk lesions can also inform revascularization strategies; for instance, deploying an additional stent to secure a high-risk lesion alongside treating a stenosis. Accurate plaque change assessments through precise registration can enhance mechanistic studies in drug development. Moreover, recognizing high-risk characteristics in IVOCT may provide valuable insights for other imaging modalities.

Deep learning holds significant potential to transform clinical care in medical imaging. It could facilitate the interpretation of IVOCT images, aiding in plaque characterization and guiding vessel preparation strategies (e.g., deciding between direct stenting and plaque modification). However, there are three major limitations that hampers its clinical adoptation. First, the deep learning data currently available for IVOCT interpretation is limited and lacks replication. Additionally, a major challenge in clinical practice is the time-intensive nature of these analyses, which often require human intervention, especially in cases with high plaque content or poor image quality. Consequently, existing software remains predominantly research-focused and labor-intensive, limiting its clinical applicability. To overcome these challenges, there is an urgent need for community-driven efforts to develop and share fully labeled public IVOCT datasets. The lack of open-source datasets not only limits model reproducibility but also raises questions about the feasibility of widespread AI adoption in this domain. Collaborative initiatives to create and maintain such datasets could accelerate innovation, ensure model reproducibility, and facilitate the integration of deep learning into routine clinical workflows. Second, the interpretability of AI models remains a critical challenge. Interpretability is essential for building clinician trust and securing regulatory approval, as it allows users to understand how AI models analyze data and make predictions. Without this transparency, clinical users may hesitate to adopt AI-driven tools, particularly in high-stakes medical applications. Techniques such as SHAP (SHapley Additive exPlanations) [141] and LIME (Local Interpretable Model-Agnostic Explanations) [142] provide valuable insights into model behaivor by highlighting the features most influential in a given prediction. Applying these methods can make AI models more transparent and understandable, thereby bridging the gap between advanced algorithms and practical clinical utility. Third, the training of AI models with diverse datasets remains a critical concern. Disparity studies emphasize the importance of accounting for sex, race, and other demographic variability, as well as differences in clinical settings, to ensure AI models are robust and generalizable. A lack of diversity in training data can result in biased models that fail to perform well across diverse populations or in varying clinical contexts. This highlights the need for strategies to obtain diverse datasets, such as multi-institutional collaborations and inclusion of underrepresented populations in data collection efforts. Addressing these challenges will improve the generalizability and robustness of AI models, ultimately enhancing their reliability and clinical utility. The recent FDA approval of AI-powered IVOCT plaque characterization software (e.g., Ultreon^™^ 2.0, Abbott Vascular, Santa Clara, CA, USA) marks a significant advancement, with its clinical use underway. The increased accuracy, accessibility, and interpretability of such software, supported by extensive datasets, will likely drive broader clinical adoption.

Advancements in software that integrates AI-driven quantitative plaque evaluations with biomechanical coronary artery analysis are poised to enhance their relevance in both research and clinical settings. The finite element method, a cornerstone in biomechanics, has already shown its effectiveness in diverse clinical applications. For instance, FFRCT (Heartflow Inc., Redwood City, CA, USA) exemplifies this by combining CFD and AI algorithms to derive FFR from CCTA for the entire coronary tree. Similarly, developing deep learning models trained on computational frameworks could expediently predict mechanical outcomes, such as stenting outcomes as a function of stent diameter and length. Furthermore, combining plaque analysis with other metrics, such as stenosis severity and OCT-derived FFR, might enhance the comprehensive cardiovascular risk assessment using IVOCT, including the progression of in-stent restenosis. Nonetheless, research demonstrating the link between plaque characteristic changes and clinical outcomes improvement is essential for incorporating these analyses into routine clinical practice.

## Supplementary Material

Table S1

This article has supplementary downloadable material available at https://doi.org/10.1109/RBME.2025.3530244, provided by the authors.
