# Supplementary material for: Computational Analysis of Intravascular OCT Images for Future Clinical Support: A Comprehensive Review
Source: IEEE Rev Biomed Eng. Author manuscript; Available in PMC 2025 Aug 12. (PMC12341395; doi:10.1109/RBME.2025.3530244)
Supplement: Table S1 [file NIHMS2097618-supplement-Table_S1.docx]

Table 1 Summary of IVOCT studies utilizing deep learning and computational analyses

| **Authors** | **Year** | **N (Images)** | **Description** | **Results** | **Env.** |
| --- | --- | --- | --- | --- | --- |
| *Image processing-based lumen/vessel wall segmentation* | | | | | |
| Chen [30] | 2018 | 100 (43,873) | Automated method for IVOCT multi-layer segmentation (LOGISMOS) | - Layer thickness correlation (*R^2^*): 0.93  - Average thickness error: 4.98±31.24 *µm* | - |
| Pociask [31] | 2018 | (667) | Lumen segmentation using a linear interpolation | - Lumen area measurement: ICC 0.97  - Lumen area difference: 0.10 *mm^2^* | MATLAB |
| Olender [32] | 2019 | 7 (724) | Anisotropic linear-elastic mesh method | - Sensitivity 90.8%, specificity 99.0%, and R^2^ 0.89 | MATLAB |
| Akbar [33] | 2019 | 40  (5,931) | Lumen segmentation using the Sobel edge detection | - Lumen segmentation similarity: *R*=0.988  - Overlapping/non-overlapping area ratios: 0.931/0.101  - OCT-derived FFR comparison: *R^2^*=0.978 | - |
| Zhang [34] | 2020 | 10  (2,283) | Lumen segmentation using a graph cut method | - Sensitivity 96.4%, specificity 98.8%, Dice 0.796  - Absolute mean difference: 4.61 pixels  - Hausdorff distance 17.1 pixels, area overlap 82.8% | - |
| Zhu [35] | 2021 | 6  (1,436) | Lumen segmentation using uniqueness of connected region | - Dice 0.946, Jaccard 0.901, accuracy 98.0% | - |
| Huang [36] | 2022 | 5  (436) | Multilayer segmentation using the edge detection and cubic spline surface fitting | - Error: IEM 1.4%, EEM 4.3%, AVD 7.0% | MATLAB |
| *Learning-based lumen/vessel wall segmentation* | | | | | |
| Abdolmanafi [38] | 2019 | 45 (5,040) | Multilayer segmentation using VGG-19 network and random forest | - Intima: sensitivity (93.0%), specificity (86.0%), accuracy (90.0%), Dice (0.990)  - Media: sensitivity (91.0%), specificity (82.0%), accuracy (87.0%), Dice (0.990) | MATLAB |
| Gharaibeh [39] | 2019 | 34 (2,640) | Lumen segmentation using SegNet and CRF noise cleaning | - Sensitivity (99.0%), specificity (99.0%), (Dice 0.990) | MATLAB |
| Balaji [40] | 2021 | 22 (12,011) | Lumen segmentation using DeepCap (preprocessing and U-Net-like architecture) | - Sensitivity (95.1%), specificity (99.7%), Dice (0.973) | Python (Pytorch) |
| *Identification of principal coronary plaques (Frame-level)* | | | | | |
| Gessert [45] | 2019 | 49 (4,000) | Two-path deep learning to classify IVOCT images as plaque or non-plaque | - Sensitivity (90.9%), specificity (92.4%), accuracy (91.7%), Dice (0.913) | Python (Tensorflow) |
| He [46] | 2020 | 18 (4,860) | ResNet-based 3D network to classify IVOCT images as calcification positive or negative | - Precision (96.9%), sensitivity (97.7%), Dice (0.961) | MATLAB |
| Niioka [47] | 2022 | 1,791 (44,947) | Diagnostic algorithm for assessing plaque vulnerability using DenseNet-121 | - Diagnostic accuracy (94%)  - AUCs: normal (0.992), stable plaque (0.952), vulnerable plaque (0.998) | - |
| Araki [49] | 2023 | 581 (237,021) | ViT-based deep learning model to detect layered plaque | - Internal validation: AUC (0.860), false positive rate (22.3), false negative rate (22.4)  - External validation: AUC (0.845), false positive rate (23.5), false negative rate (24.0) | Python (Scikit-Learn) |
| *Identification of principal coronary plaques (A-line)* | | | | | |
| Kolluru [50] | 2018 | 48  (4,469) | A-line classification using a shallow CNN and CRF noise cleaning | - Sensitivity: fibrocalcific (80.0%), fibrolipidic (95.0%)  - Specificity: fibrocalcific (85.0%), fibrolipidic (92.0%) | MATLAB |
| Cheimariotis [51] | 2021 | 33  (183) | A-line classification using the ARC-OCT and shallow CNN models | - Overall accuracy: 83.5% for transformed images | MATLAB |
| Prabhu [53] | 2019 | 59  (6,996) | A-line classification using a comprehensive set of handcrafted plaque features and SVM | - Sensitivity: fibrolipidic (94.5%), fibrocalcific (74.8%)  - Specificity: fibrolipidic (87.3%), fibrocalcific (95.3%) | MATLAB |
| Lee [54] | 2020 | 49 (6,556) | A-line plaque characterization using a hybrid deep convolutional and hand-crafted features | - Fibrolipidic plaque: sensitivity (84.8%), specificity (97.8%), Dice (0.887)  - Fibrocalcific plaque: sensitivity (91.2%), specificity (96.2%), Dice (0.719) | MATLAB |
| *Identification of principal coronary plaques (Pixel-wise)* | | | | | |
| Zhang [55] | 2019 | (18,600) | Plaque segmentation using CNN and random walk algorithm | - Jaccard: fibrotic plaque (0.876) and lipid/calcified plaques (0.864)  - Average symmetric contour distance: 0.38 *mm* | MATLAB |
| Gharaibeh [39] | 2019 | 34  (2,640) | Calcification segmentation using SegNet | - Sensitivity (85.0%), specificity (99.0%), Dice (0.730) | MATLAB |
| Lee [56] | 2019 | 55  (4,892) | Plaque segmentation using novel preprocessing, SegNet, and CRF noise cleaning | - Lipid: sensitivity (87.4%), specificity (89.5%)  - Calcification: sensitivity (85.1%), specificity (94.2%) | MATLAB |
| Li and Jia [57] | 2019 | (2,000) | Vulnerable plaque segmentation using deep residual U-Net | - Precision (94.3%), sensitivity (91.2%), accuracy (93.3%), IoU (0.855) | - |
| Lee [58] | 2020 | 70 (12,551) | Two-step deep learning method for characterizing calcified plaque | - Sensitivity (86.2%), precision (75.8%), Dice (0.781) | MATLAB |
| Tang [59] | 2023 | 14  (2,388) | Three-step framework for plaque segmentation using U-Net and self-attention ResNet | - Sensitivity: calcium (78.4%), lipid (80.2%)  - Dice: calcium (0.718), lipid (0.605) | Python (Pytorch) |
| Ren [60] | 2023 | 7  (490) | Two-step plaque characterization using level-set and dense-block-Segnet | - Sensitivity: fibrous (92.8%), lipid (91.8%), calcification (91.8%) | Python (Pytorch) |
| Chu [61] | 2021 | 509 (11,673) | Plaque segmentation using a deep convolutional network with a U-Net architecture | - Dice: fibrous (0.906), calcium (0.848), lipid (0.772) | - |
| Lee [62] | 2023 | 34  (2,723) | OCTOPUS software for coronary plaque characterization | - 23% of frames needed any manual touch up for detailed calcification labeling | MATLAB |
| Kolluru [63] | 2021 | 60  (3,741) | Comparison of varying number of training samples derived from equally spaced sub-sampling and deep learning-based clustering | - All: F1 (0.74), Precision (77%)  - One-half equal spacing: F1 (0.72), Precision (74%)  - One-half clustered: F1 (0.73), Precision (76%) | MATLAB |
| Abdolmanafi [38] | 2019 | 45  (5,040) | Lesion detection/classification using three CNN networks and random forest classifier | - Sensitivity: fibrosis (94.0%), calcification (84.0%)  - Specificity: fibrosis (96.0%), calcification (95.0%) | MATLAB |
| Bian [64] | 2020 | 46 (12,420) | Semi-automated method for plaque identification using texture features | - Average accuracy (90.3%) | - |
| Sun [65] | 2022 | 83 (29,914) | Detection of plaque erosion using Mask R-CNN and SVM | - Sensitivity (80.0%), precision (73.4%), AUC (0.707) | Python (Tensorflow) |
| *Segmentation of microscopic coronary plaques* | | | | | |
| Abdolmanafi [38] | 2019 | 45  (5,040) | Macrophage detection using three pre-trained networks and random forest classifier | - Sensitivity (89.0%), specificity (97.0%), accuracy (92.0%) | MATLAB |
| Rico-Jimenez [69] | 2019 | 28  (252) | Macrophage detection by calculating normalized-intensity standard deviation | - Sensitivity (88.0%), specificity (88.0%) | MATLAB |
| Guo [70] | 2019 | 9  (1) | Lipid and fibrous cap segmentation using texture features and SVM | - Difference of cap thickness measurements compared with manual: M1 (15.1%), M2 (10.3%) | - |
| Lee [71] | 2022 | 41  (4,360) | Lipid and fibrous cap segmentation using DeepLab-v3 plus and dynamic programming | - Required a significant modification for only 5.5% frames  - FC thickness bias: 4.2±14.6 *µm* | MATLAB |
| Chu [61] | 2021 | 509 (11,673) | U-shaped encoder-decoder deep learning model with pseudo-3D input | - Cholesterol crystals: precision (54.3%), sensitivity (50.8%), Dice (0.525)  - Macrophage: precision (42.9%), sensitivity (56.8%), Dice (0.489)  - Microchannel: precision (59.8%), sensitivity (60.4%), Dice (0.601) | - |
| Lee [72] | 2024 | 227  (32,000) | Deep learning segmentation of fibrous cap using novel preprocessing and SegResNet | - Dice (0.846 for internal testing and 0.816 for external validation)  - Thickness measurement error: ~3.0 *µm* | Python (Pytorch) |
| Lee [73] | 2022 | 79  (8,403) | Two-step microchannel segmentation using DeepLab v3 plus and shallow CNN | - Candidate classification: sensitivity (99.5%), specificity (98.8%), accuracy (99.1%))  - Segmentation: sensitivity (87.7%), specificity (99.8%), Dice (0.73) | MATLAB |
| *Stent analysis* | | | | | |
| Wang [88] | 2015 | 72  (8,000) | Stent strut detection using a Bayesian network and graph search algorithm | - Recall: 91.0%, precision: 84.0% | MATLAB |
| Lu [89] | 2019 | 80  (7,125) | Stent strut detection/classification using hand-drafted features and SVM | - Strut classification: sensitivity (94%), specificity (90%)  - Strut to lumen distance error: 0.1±2.9 *µm* | MATLAB |
| Jiang [90] | 2020 | -  (165) | Detection of metallic stent struts using YOLOv3 and R-FCN networks | - Stent strut detection: precision (96.4%), sensitivity (96%) | - |
| Wu [91] | 2020 | 230  (31,780) | U-shape-like deep learning model for stent strut detection | - Stent strut segmentation: Dice (0.907), Jaccard (0.838)  - Stent strut detection: precision (94.3%), sensitivity (94.0%) | - |
| Lau [92] | 2021 | 51  (17,799) | U-Net combined with MobileNetV2 and DenseNet121 to segment metal stents and bioresorbable scaffolds | - BVS segmentation: Dice (0.860)  - Metal stent segmentation: precision (92.0%), sensitivity (92.0%) | Python (Tensorflow) |
| Lu [94] | 2020 | 292  (109,500) | OCTivat-Stent software for stent analysis | - CCC of automatically measured stent region: 0.970  - Strut classification: uncovered (82%), covered (99%) | MATLAB |
| *OCT-derived FFR* | | | | | |
| Yu [97] | 2019 | 125  (-) | OctPlus software for automated computation of OFR from IVOCT | - Detection of FFR<0.8: sensitivity (87.0%), specificity (92.0%), accuracy (90.0%) | - |
| Huang [98] | 2020 | 212  (-) | External validation of OctPlus OFR | - Detection of FFR<0.8: sensitivity (86.0%), specificity (95.0%), accuracy (92.0%)  - Agreement with wire-based FFR: *r*=0.87, ICC=0.87 | - |
| Gutiérrez-Chico [99] | 2020 | 76  (-) | External validation of OctPlus OFR | - Detection of FFR<0.8: sensitivity (92.0%), specificity (93.0%), accuracy (93.0%)  - Median time to obtain OCT-FFR: 1.07 min | - |
| Cha [100] | 2020 | 125  (-) | Prediction of OCT-based FFR using random forest model | - Detection of FFR<0.8: sensitivity (100%), specificity (92.9%), accuracy (95.2%)  - Agreement with wire-based FFR: *r*=0.853, MAE=0.04 | - |
| *IVOCT pullback registration* | | | | | |
| Gharaibeh [101] | 2020 | 11  (5,940) | Registration of pre- and post-stenting pullbacks using the cross-correlations of 1D graphs | - Accuracy: <1 frame interval (±200 *µm*) | MATLAB |
| *Computer simulation with FEA and CFD* | | | | | |
| He [102] | 2020 | 1  (300) | Effects of multiple stents with and without overlap using patient-specific FEA | - Stent overlap led to greater lumen gain, but also poses a higher risk of tissue damage and restenosis. | Mimics |
| Dong [103] | 2021 | 1  (131) | Influence of calcification features on stent expansion using patient-specific FEA | - Stent expansion was strongly associated with the calcification angle and area. | Mimics |
| Dong [104] | 2021 | 1  (540) | Impact of post-dilation parameters to enhance stent expansion using patient-specific FEA | - A balloon at a higher inflation pressure could safely improve the lumen area. | Mimics |
| Gamage [105] | 2022 | 1  (540) | Hemodynamic alternations following stenting and post-dilation using patient-specific CFD | - Malapposed struts induced much higher shear rate, flow disturbances, and lower time-averaged wall shear stress. | SpaceClaim / ICEM CFD |
| Gamage [106] | 2022 | 1  (375) | Influence of side branches on FFR evaluation using patient-specific CFD | - The upstream side branch had a minimal effect on FFR, whereas a downstream branch resulted in a lower FFR. | SpaceClaim / ICEM CFD |
| *IVOCT plaque characteristics used to refine CCTA analyses* | | | | | |
| Kolossváry [116] | 2019 | 44  (-) | Plaque and radiomic features from CTA to predict OCT-defined TCFA | - AUC: low attenuation plaque (0.66), radiomics (0.80) | Python  (Scikit-learn) |
| Kim [117] | 2023 | 30  (-) | PCAT radiomics features to predict IVOCT-defined vulnerable plaque (e.g., TCFA) | - TCFA: AUC (0.78±0.13)  - Microchannel: AUC (0.89±0.09) | Python (Pytorch) |
| Cao [118] | 2023 | 41  (-) | Comparison of plaques (calcified, non-calcified, and mixed) between CTA and OCT | - Sensitivity (92.0%), specificity (98.0%), accuracy (93.0%) | - |
| *Clinical research – Stenting outcome prediction* | | | | | |
| Fujino [28] | 2018 | 261  (-) | OCT-based calcium scoring system to predict stent underexpansion. | - Predictability for SEI<70%: AUC (0.86) | JMP |
| Gharaibeh [29] | 2023 | 110  (-) | Prediction of post-stent lumen area and SEI using hand-crafted plaque features. | - Lumen area: RMSE (0.04±0.02 *mm^2^)*, *r*=0.94±0.04  - Detection of SEI<80%: AUC (0.85±0.02) | MATLAB |
| *Clinical research – MACE prediction* | | | | | |
| Montone [119] | 2020 | 153  (-) | Correlation between OCT-defined macrophage infiltrates and MACE. | - Plaque erosion with macrophage infiltration for predicting MACE: HR 3.0, 95% CI 1.1-8.0, *p*=0.034 | SPSS |
| Zhao [120] | 2021 | 434  (-) | Prognostic significance of TCFA and residual syntax score (rSS) for MACE. | - MACE predictability: AUC (0.816, 95% CI 0.765-0.860) | SPSS |
| Kim [82] | 2022 | 1,290  (-) | Post-stenting IVOCT findings to predict adverse events (DoCE and MSE). | - DoCE: MSA (HR 1.2, 95% CI 1.0-1.4, *p*=0.045)  - MSE: Malapposition (HR 6.1, 95% CI 1.9-20.0, *p*=0.003) | SPSS/R |
| *Clinical research – Neo-atherosclerosis prediction* | | | | | |
| Lee [121] | 2022 | 180  (-) | Association between IVOCT plaque characteristics and neo-atherosclerosis | - Predictor for neo-atherosclerosis: FC surface area (OR 1.4, 95% CI 1.1-1.8, *p*<0.05, AUC 0.901) | R |
| *Clinical research – TVF prediction* | | | | | |
| Chen [122] | 2022 | 512  (-) | Clinical and morphological characteristics to predict target lesion failure. | - TVF predictors: lipidic neo-atherosclerosis (HR 2.1, 95% CI 1.4-3.1, *p*<0.001) and female sex (HR 1.6, 95% CI 1.1-2.3, *p*=0.03) | SPSS |
| de Filippo [123] | 2023 | 1,801  (-) | Clinical and intracoronary imaging factors to predict target vessel failure | - TVF predictor: total stent length (HR 1.01, 95% CI 1.00-1.02, *p*<0.05)  - TVF protector: use of intracoronary imaging (HR 0.35, 95% CI 0.12-0.82, *p*<0.05) | SPSS |
| Kang [124] | 2023 | 2008  (-) | Comparison of clinical outcomes between OCT-guided and IVUS-guided PCI | - The incidence of TVF of OCT and IVUS groups: 2.2% and 3.7%, respectively (*p*=0.047) | SAS |
| *Clinical research – CAV prediction* | | | | | |
| Pazdernik [125] | 2018 | 50  (-) | Automated 3D IVOCT vessel wall analysis to quantify CAV | - Significant reduction in the mean luminal area (*p*<0.05) and progression in mean intimal thickness (*p*<0.05) | R |
| Chih [126] | 2023 | 82  (-) | OCT imaging predictors of early CAV progression | - Median intimal volume increase: 42%  - Predictors of CAV progression: male sex, fibrotic plaque, and index of microcirculatory resistance | SAS |
| Jensen [127] | 2023 | 114  (-) | Prognostic potential of OCT-derived measurements for CAV | - CAV predictors: LAD derived intima/media ratio (HR 3.4, 95% CI 1.3-8.6, *p*=0.03), lumen/intima ratio (HR 2.8, 95% CI 1.1-7.1, *p*=0.03) | STATA |
| Cote [128] | 2018 | 17  (-) | Comparison of intimal/medial thickness and cross-sectional area with LV strain | - LV strain to intima thickness (*r*=-0.497, *p*=0.042)  - LV strain to media thickness (*r*=-0.503, *p*=0.039) | SPSS |
| McGovern [129] | 2019 | 110  (-) | OCT findings to quantify CAV | - Median intima thickness: previous/current rejection (0.12, IQR 0.09-0.17) and no rejection (0.09, IQR 0.08-0.13) | STATA |
| Orban [130] | 2020 | 45  (-) | Comparison of OCT findings between pediatric and adult HTx patients | - ≥ 5years intima/media: adult (2.3, IQR 1.6-3.6) vs. pediatric (5.4, IQR 2.8-13.4)  - >5 and ≤ 10 years intima thickness: adult (0.4, IQR 0.3-0.7) vs. pediatric (0.2, IQR 0.1-0.3) | SPSS |
| Pazdernik [131] | 2020 | 116  (-) | Automated 3D IVOCT vessel wall analysis to quantify CAV | - Significant reduction in the mean luminal area (*p*<0.05) and progression in mean intimal thickness (*p*<0.05) | R |
| Pazdernik [132] | 2021 | 104  (-) | Automated analysis of luminal, intimal-layer, and medial-layer using LOGISMOS | - Significant reduction in the mean luminal area (p<0.001) and progression in mean intimal thickness (p<0.001) | R |
| Chahal [133] | 2020 | 45  (-) | Association of neo-vessels within the intima with intimal thickening and co-incident CAV | - CAV predictors: neo-vessels (OR 12.8, 95% CI 3.1-53.2) | SPSS |
| Reddy [134] | 2021 | 55  (-) | Comparison of severe CAVs detected with angiography, IVUS, and OCT | - Severe CAV detection with MIT > 0.25 *mm*: sensitivity (86.7%), specificity (74.3%) | R |
| Rafique [135] | 2021 | 81  (-) | Short-term effects of high-intensity interval training on CAV as assessed by OCT | - Intima area: high intensity (0.6±1.2 *mm^2^*) and moderate intensity (0.3±0.6 *mm^2^*) | STATA |
| Takahashi [136] | 2023 | 35  (-) | Prognostic role of early qualitative assessment using OCT for CAV | - Predictor of clinical events: OCT-defined plaque morphology (HR 4.6, 95% CI 1.5-13.9, *p*=0.008) | SPSS/R |
